# Assessment of mold contamination and physicochemical properties of crude palm oil sold in Jos, Nigeria

**DOI:** 10.1002/fsn3.393

**Published:** 2016-06-15

**Authors:** Chuks K. Odoh, Tarfen Y. Amapu, Ikechukwu P. Orjiakor, Paul E. Martins, Benedict T. Seibai, Uche K. Akpi, Chinyere S. Ugwu, Nathaniel I. Lerum, Amechi S. Nwankwegu

**Affiliations:** ^1^ Department of MicrobiologyUniversity of NigeriaNsukkaNigeria; ^2^ Department of Science Laboratory TechnologyUniversity of JosJosNigeria; ^3^ Department of MicrobiologyUniversity of BeninBeninNigeria; ^4^ Department of Agricultural ExtensionUniversity of NigeriaNsukkaNigeria; ^5^ Department of Applied Microbiology and BrewingNnamdi Azikiwe UniversityAwkaNigeria

**Keywords:** Carcinogenic, contamination, mold, palm oil, physicochemical

## Abstract

Due to increasing reports on poor‐quality palm oil in the market, there has been a continual decrease in demand and revenue cum product rejection of palm oil sold in Jos, Nigeria. Hence, the significance of this work aims to moderate the microbial and physical qualities of crude palm oil sold in different major markets in order to increase revenue through quality control and quality assurance protocols. The intent is to create awareness among government monitoring agencies, buyers (exporters and importers), and to promote standard processing procedures among manufacturers. The study was carried out to ascertain the levels of mold contamination and physicochemical properties of crude palm oil sold in Jos and its environ. A total of 90 samples were collected in sterile containers. Molds were isolated and identified using standard identification procedures. The physicochemical properties: free fatty acids (FFA), peroxide value (PV), iodine value (IV), moisture content (MC), and impurity level were determined. The assessment of mold isolated from the study sites recognized some life‐threatening genera capable of producing carcinogenic toxins. *Candida* sp. (51%) had the highest percentage of occurrence followed by *Aspergillus* sp. (45%) while *Fusarium* sp. (3%) was the least occurring mold. The mean mold count for all the palm oil samples ranges from 3.18 × 10^4^ (cfu/mL)–4.56 × 10^4^ (cfu/mL). Physicochemical findings showed that the average FFA (3.43–6.88%), PV (6.96–13.63 mEq/kg), IV (39.7–67.5 wijs), and MC (0.44–0.72%) values were within the SON acceptable limit, except the impurity level (0.28–0.44%) which was higher than the acceptable SON range in all the sites. There was a significant difference (*P* ≤ 0.05) in the mold and physicochemical properties of crude palm oil in almost all the samples analyzed when compared to both local and international permissible limits of the tested parameters recommended for edible palm oil. There was compliance between the permissible limits (local and international) of the physicochemical values of the parameters we tested for in the edible palm oil except the impurity level, while the mold count did not meet (was higher) with the required permissible limit of the SON mold count.

## Introduction

Palm oil is one of the most consumed edible oils within the tropics. In Nigeria, it forms the basic food ingredient of every household. It is a product of oil palm tree (*Elaeis guineensis*), an ancestral cash crop tree of African origin. It is believed to have spread to various countries by farmers who practiced shifting cultivation. History has shown that human use of palm oil may be dated as far back as 5000 years in West Africa (Kiple and Conee 2000; Poku, 2002). All over the globe, palm oil has become one of the leading sources of income especially in Africa and Asia, with a special reference to Malaysia where it has become the most relied on source of revenue for the government. In tropical Africa, South‐East Asia and parts of Brazil, palm oil is a common cooking ingredient, while in commercial industries, it is used for manufacturing soaps, washing powder, and other products (Bellis 2005). Since 2006, palm oil has become the world's most important edible oil (ISTA 2007). It is also a source of energy and fat deposits which insulate the body against loss of heat and also protects the vital organs against mechanical injury (Baku et al. 2012). Palm oil is an important food source to humans because it supplies essential fatty acids such as linoleic and arachidonic acid and also contains large amounts of tocotrienol which is a part of the vitamin E family (Bonnie and Choo 2000).

In Nigeria, the demand for palm oil has been on the rise due to the rising population and the need to meet international market demand. This has necessitated an increase in the manual and traditional process of production which is predominantly carried out by villagers with little or no knowledge of aseptic techniques. There has been speculation that the potential harmful effects of unrefined traditionally processed palm oil could outweigh its nutritional benefits since these oils could be containing some components that enhance numerous reactions involved in the degradation of the product (Tagoe et al. 2012). Microbial contamination may occur during handling, leading to changes in the quality and chemical composition (Okpokwasili and Molokwu 1996) which is characterized by rancidity, acidity, soapiness and other odd flavor, especially when triglycerides of the oil are hydrolyzed by lipolytic fungi (Larry 1987).

Molds are one of the possible microbial contaminants of crude palm oil. Molds are a diverse group of fungal species, with hyphal growth resulting in discoloration and a fuzzy appearance, especially when found on food (Morgan 2012). They are ubiquitous and their spores are dispersed through air and water bodies. Intake of spores can lead to health challenges such as allergic reactions, respiratory problems, and the release of toxins which could be fatal (Empting 2009). As these organisms find their way into palm oil, they proliferate due to the moisture content of the oil (Pitt and Hocking 2009). Reports indicate that these organisms could survive at extreme conditions of temperature, pressure, high salinity, and even at low water activity by spore formation (Malloch 1981). Thus, the possibility of mold contamination of crude palm oil and its transmission to humans is understandable.

Recently, Goudoum et al. (2015), reported that temperature and other storage conditions have a direct effect on the physicochemical qualities of crude palm oil. In their observation, they highlighted that physicochemical qualities are the major properties that determine the quality of oil and help describe its present condition. In a separate study, it was understood that storage of crude palm oil over a long time period could be one of the possible causes of fast deterioration of the essential chemical qualities of the oil such as: FFA, PV, and IV (Ekwenye 2005). Most merchants in oil business store their products for a very long time as they await rise in market price. Hence, the dearth of published article and the quest to understand in detail, the levels of mold contamination, physical and chemical properties of crude palm oil sold in Jos, Nigeria, led to this research work.

## Materials and Methods

### Study area

This study was carried out in Jos, Plateau State, North Central Nigeria (Latitude 80^o^24′N, Longitude 80^o^32′ and 100^o^38′E).

### Sampling area

Crude palm oil samples were collected from three popular markets within the metropolis. These include Bukuru and its environ (site A), Terminus market/Bauchi road (site B), and Faringada/Gadabiu (site C). A total of 90 samples were collected; 30 from each location. These were collected at random from 30 palm oil sellers at each collection site.

### Sample collection

About 200 mL of palm oil samples were collected aseptically into sterile glass bottles, capped and labeled properly. The samples were placed in a polythene bag and transported immediately to the Microbiology (Food/Environmental Laboratory) department in University of Nigeria, Nsukka for analysis.

### Sample preparation

A stock solution was prepared by diluting 10 mL of the crude palm oil samples into a sterile 90 mL oil emulsifier (Tween 80). A quantity of 1 mL of the stock solution was used for serial dilution using 9 mL of sterile distilled water. At the end of the dilution process, 0.1 mL of the last diluents were inoculated onto sterile Sabouraud Dextrose agar (HKM PRC) plates in triplicate and incubated at room temperature. Streptomycin (50 *μ*g/mL) was added to the molten agar to prevent bacterial growth prior to incubation. Mold counts were taken after 3–7 days. For the purposes of identification, mold colonies were picked with a sterile inoculating loop and subcultured on freshly prepared Sabouraud Dextrose agar (SDA). The molds were purified by streaking on the same agar. Pure isolates were stored in SDA slants at 2–8^°^C prior to macroscopic and microscopic identification using standard procedures.

### Isolation and identification of molds

Macroscopic identification was done using the culture characteristics and appearances of the isolates. General features of molds were looked out for following standard mycological identification process (Beneke and Rogers 1980). Also, strict microscopic identification procedures were adhered to as described by Enemuor et al. (2012), by picking a small portion of the culture onto a clean sterile slide containing a drop of lactophenol blue with the aid of a sterile needle. This was done after the ethanol used for decontaminating the slide had evaporated. The slides were covered with a cover slip and viewed under the light microscope (Motic B1‐220A, PRC). The microscopic characteristics of hyphae were noted and compared with those in the reference manual of Barnett and Hunter (1972), for proper identification.

### Physicochemical properties of palm oil

Free fatty acids (FFA) were measured using British Standard Method (1976). This was done by measuring 50 mL of 95% ethanol into 5 g of palm oil sample contained in a 250 mL conical flask. After homogenizing for a while, it was heated until the oil dissolved completely. Six drops of phenolphthalein indicator was added and the mixture titrated with 0.1 mol/L KOH until an end point coloration (pink, red or orange). The values obtained from the findings were later calculated in percentage.

$$\text{Free Fatty Acids}\left(\%\right)=\frac{\text{mL of KOH}\times \text{Molarity}\times 25.6}{\text{Weight of palm oil}(g)}$$

Peroxide value (PV) was determined by titrating potassium iodide solution of the oil with an aqueous solution of sodium thiosulphate using starch as an indicator (Aletor et al. 1990). In this method, 5 g of oil was weighed into a 250‐mL conical flask. A mixture of glacial acetic acid and trichloromethane chloroform was added in the ratio of 3:2. About 0.5 mL of saturated (144 g per 100 g of distilled water) potassium iodide solution was also added. After shaking, about 30 mL of water was added. The solution was titrated with 0.1 N sodium thiosulphate until yellow color was almost unseen. About 0.5 mL of starch indicator was added to the titration process while shaking vigorously until the blue–black color disappeared. A blank sample devoid of palm oil samples was also analyzed using the same procedure. The differences between the two titer values of sodium thiosulphate utilized were determined.

$$\text{Peroxide value (Meq Peroxide/kg)}=\frac{\text{S‐B}\times M\times 1000}{\text{Sample weight}(g)}$$

where: S = Sample titer (mL)

B = Blank titer (mL)

M = Molarity of thiosulphate

For the determination of impurity level, as described by Ngando et al. (2011), about 10 mL of the crude oil sample was weighed into a beaker containing excess hexane (20–30 mL), heated at 105°C, and filtered. The residue on the filter paper was further washed with hexane. The filtered sample was allowed to cool in a desiccator and the residual weight or particles obtained on the filter paper were calculated as percentage impurity.

$$\text{Impurity level, IL}=\frac{A\times 100}{\text{AB}}$$

where: A = weight of desiccated residual particles

AB = total weight of the sample before treatment

Iodine value (IV) was determined using Wijs solution method according to Food Safety and Standard Authority of India (2012).

The moisture content of the palm oil samples was determined using the British Standard Method (1976). A quantity of 5 g of the palm oil was weighed into a crucible and placed in an oven for about 2 h at 110°C. It was allowed to cool in a desiccator and its final weight determined.

$$\text{Percentage moisture}=\frac{W_{2}-W_{3}\times 100}{W_{2}-W_{1}}$$

Weight of crucible (empty) = W_1 _g

Weight of crucible + palm oil before drying = W_2 _g

Weight of oil = (W_2_−W_1_) g

Weight of crucible + palm oil after drying = W_3 _g

The moisture content was obtained by mass difference = (W_2_−W_3_)g

### Statistical analysis

The data obtained were analyzed at *P* ≤ 0.05 (ANOVA) using Statistical Package for Social Sciences (SPSS, Armonk, NY, USA) version 20.

## Results

Table 1 Comparison of the physicochemical properties and mold counts of palm oil with the standard requirements of crude palm oil.

**Table 1:** Comparison of the physicochemical properties and mold counts of palm oil with the standard requirements of crude palm oil

Parameters	PORAM (crude palm oil)	PORAM (refined oil)	SON permissible limit	Site A	Site B	Site C
Free fatty acid (%)	5 (max)	0.10	3–5	4.70 ± 0.75	6.88 ± 0.97	3.43 ± 0.40
Iodine value (wijs)	56 (min)	50–55	43–53	39.77 ± 0.46	67.55 ± 0.58	48.79 ± 0.61
Peroxide value (mEq/kg)	a	2	10	7.90 ± 0.25	13.63 ± 0.57	6.96 ± 0.45
Moisture contents (%)	0.50	0.25 (max)	0.29 (max)	0.57 ± 0.03	0.72 ± 0.03	0.44 ± 0.04
Impurity level (%)	0.50	0.25(max)	0.2	0.44 ± 0.04	0.28 ± 0.02	0.35 ± 0.02
Mold count	a	a	2 × 10^4^ (cfu/mL)	4.04 ± 0.17	4.56 ± 0.30	3.18 ± 0.27

PORAM: Palm Oil Refiners Association of Malaysia (2005), SON: Standard Organization of Nigeria (2000).

a No value. Samples values are the mean representation of triplicate sample collection per site.

**Figure 1:**
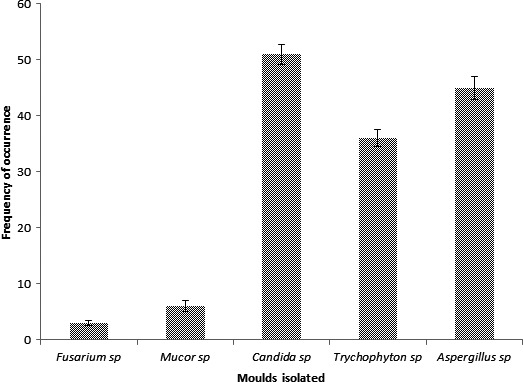
Frequency occurrence of mold in the total crude palm oil samples obtained from Jos and its environ.

Figure 1 represents an array of molds isolated from the total samples obtained within Jos and its environ. *Candida* sp. and *Aspergillus* sp. were dominant while *Fusarium* sp. and *Mucor* sp. were found to have the least frequency occurrence among the total samples. The number of different molds isolated in the total samples were: Candida sp 51 (56.6%), *Aspergillus* sp. 45 (50%), *Trichophyton* sp. 36 (40%), *Mucor* sp. 6 (6.66%), and *Fusarium* sp.3 (3.33%).

**Figure 2:**
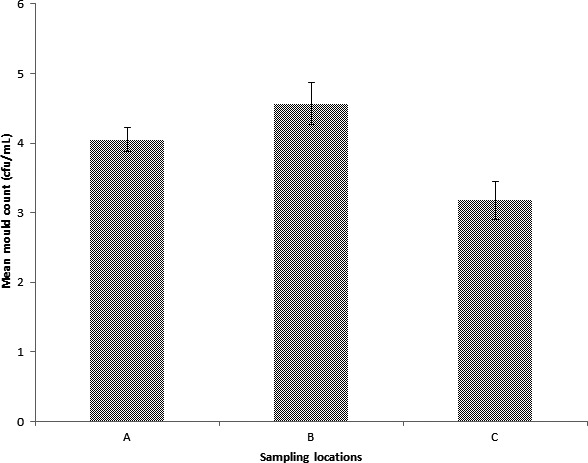
The mean mold count of crude palm oil samples obtained from Jos and its environ.

Figure 2 illustrates the mean mold count of the crude palm oil obtained from three different study sites within Jos. The sample from site B had the highest mold count, followed by that of site A, while the least mold counts were obtained from site C.

## Discussion

The result obtained in this study showed that palm oil samples from the study sites has reasonable amount of mold‐producing genera. The organisms isolated from the palm oil samples include: *Aspergillu*s sp.*, Candida* sp.*, Fusarium* sp., *Muco*r sp.*,* and *Trichophyton* sp. which are similar to those obtained in previous works by Okechalu et al. (2011) and Enemuor et al. (2012). There are indications that these molds are able to survive the anaerobic nature of the palm oil through lipase production and spore formation (Reddy et al. 2009). These organisms are believed to aid fast deterioration of palm oil as well as toxin production (aflatoxin) which could cause health challenge when consumed. The mold counts in the palm oil samples were found to be significantly higher (*P* ≤ 0.05) than the recommended permissible limit (2 × 10^4 ^cfu/mL) for edible oil. These organisms can be life threatening as they can survive high temperature during cooking or UV radiation, as they form spores (Doyle et al. 1997; Ekwenye 2005).

PV is an indicator which defines the onset of oxidative change in oil. This change in palm oil is initiated mostly by several biotic and abiotic conditions such as microorganism, storage temperature, light intensity, and oxygen. The result obtained from this work implicated the crude palm oil obtained from site B to have the highest PV reading. This is in agreement with the work of Okpokwasili and Molokwu (1996), with their report showing that microorganisms are the major cause of chemical change in palm oil. From our result, PV obtained from site A and C (7.9 mEq/kg, 6.96 mEq/kg), respectively, fall within the permissible range 10 mEq/kg (SON, 2000) for edible palm oil; and in tandem with the work of Agbaire (2012), who reported a range of 7.80–8.40 mEq/kg. Although the values obtained from the two sites fall within the acceptable standard range, they are still on a high side especially when compared to the PORAM standard. This is evidence of the early rancidity and primary oxidation due to lipid degrading enzymes like peroxidase and lipoxygenase (Onyeka et al. 2005).

Basically, FFA is the widely used method for examining the quality of palm oil. They are expressed as palmitic acid and must not exceed the 5% permissible value recommended for edible oil. The FFA value obtained from site A (4.7%) and C (3.4%) in this research is in consonance with the result of Okechalu et al. (2011), where a range of 2.67–4.20% was reported. Contrary to this, samples from site B showed high level of FFA above the standard. This may be due to decomposition of glycerides by the mold which was found to be highest in palm oil obtained from that location, or maybe due to exposure of the palm oil to sunlight by the sellers of these oil in open market places. The values obtained from site B are in agreement with the work of Aletor et al. (1990), Orji and Mbata (2008), and Ngando et al. (2011), who in their separate researches identified PV of locally processed palm oil to be higher than the permissible standard range.

IV is an essential quality of palm oil that determines how fresh or safe an oil is for consumption based on the level of rancidity. According to Ekwenye (2005), the higher the level of IV, the more readily the palm oil becomes rancid. Some of the crude palm oil analyzed in this research showed low level of IV which were mostly within the acceptable limit, with the least values observed in site A (39.7 wijs). There was a significant difference (*P* ≤ 0.05) from the values obtained from the three locations, indicating that they may have been subjected to different environmental conditions. The results obtained are comparable to the results of Udensi and Iroegbu (2007), Okechalu et al. (2011), and Agbaire (2012), where a relatively higher value was reported. The high level of IV from site B illustrates level of unsaturation and susceptibility to oxidative rancidity which could be due to handling, prolonged storage, and other odd activities of the merchants.

Reports have suggested that impurities get into crude palm oil during the final extraction and clarification procedures (Poku 2002; Orji and Mbata 2008). Unrefined palm oil is also believed to have acquired these impurities due to handling. Palm oil analyzed in this work showed a high level of impurities when compared with the SON permissible limit. However, they are still within the permissible limit of the PORAM standard for crude palm oil. It could be deduced that the significant amount of impurities obtained in this research could be linked to the kind of storage facilities most oil marketers use during storage (hauding) as they await rise in market price. These storage facilities are mostly metallic in nature and thus rust with time, thereby releasing their particles into the oil. Furthermore, this could be deposited by air or breeze as most of the oil were sold/purchased openly by sellers/buyers. The impurity level obtained from this research (0.28–0.44%) is in agreement with the work of Aletor et al. (1990) and Enemuor et al. (2012), where reports of impurity levels of locally processed palm oil was 0.1–0.73%, and contrary to the reports of Onwuka and Akaerue (2006), who identified traditionally processed palm oil impurity content as 1.6%.

The moisture content of all the oil samples was higher than the SON and PORAM standards for crude palm oil. The values obtained from this research could be linked to processing method during production and the geographical location, since Plateau State is characterized by constant rainfall and relatively high humidity. Orji and Mbata (2008), and Enemuor et al. (2012), also identified accidental wetting or careless handling of the oil as a cause of increase in moisture content as well as inadequate boiling of the pure oil by local producers to reduce the moisture content. Furthermore, the values obtained (0.44–0.72%) are, however, lower than those reported by Aletor et al. (1990) and Okechalu et al. (2011), which were significantly higher (0.43–1.80% and 1.09–1.27%, respectively) than the local and international permissible limit for crude palm oil.

## Conclusion

About 70% of the total number of palm oil samples obtained within Jos, North Central Nigeria and its environ did not meet the recommended standard stipulated by SON and PORAM for crude palm oil. Therefore, it is reasonable to conclude that the quality of the palm oil tested was altered. This was demonstrated in the physicochemical analysis which is an indicator of the level of adulteration, rancidity, deterioration, storage condition, and handling process during production. Undoubtedly, molds isolated could present health challenges (as a result of spores and toxin production) to individuals who consume this product unrefined or without further treatment. Hence, there is need for the government to enforce quality control assurance procedures wherever palm oil is being processed for commercial purposes. The government should also create a scheme that tests and confirms the benchmark of the palm oil before their sale in market.

## Conflict of Interest

None declared.
